# Genomic characteristics and tracing analysis of an acute gastroenteritis outbreak associated with rotavirus C in a boarding high school

**DOI:** 10.1016/j.imj.2026.100276

**Published:** 2026-07-31

**Authors:** Dawei Gao, Zhichao Chen, Shunyi Zhang, Lulu Zhang, Hongwei Chang, Junli Chen, Yuanyuan Shao, Shukun Yu, Chong Liu

**Affiliations:** a Lu’an Center for Disease Control and Prevention, Key Discipline of Preventive Medicine in Anhui Province, Lu’an, 237000, China; b Huoshan County Center for Disease Control and Prevention, Lu’an, 237200, China; c Wuhan Dongxihu District Center for Disease Control and Prevention, Wuhan, 430040, China

**Keywords:** Rotavirus C, Boarding high school, Acute gastroenteritis outbreak, Molecular tracing, Evolutionary analysis

## Abstract

**Background:**

Rotaviruses are major pathogens of childhood acute gastroenteritis, dominated by rotavirus A (RVA). Outbreaks caused by human rotavirus C (RVC) are rarely reported, and relevant genomic data remain scarce. This genomic investigation of an RVC outbreak improves our understanding of viral diversity and transmission dynamics.

**Methods:**

We performed epidemiological surveys, nucleic acid testing and whole-genome sequencing (WGS) on specimens from a 2025 RVC-associated gastroenteritis outbreak at a Chinese boarding high school. Sequence alignment, phylogenetic and molecular tracing analyses were conducted to explore RVC evolution via point mutation, segment reassortment and genomic recombination.

**Results:**

This typical point-source campus outbreak was linked to an indoor student gathering matching the incubation period of RVC. Thirteen RVC FX strains were recovered from 11 rectal swabs and two vomitus samples. Their viral protein (VP) 4 and VP7 sequences shared high homology with Russian reference strains, carrying distinct amino acid variations. No segment reassortment or recombination was detected in VP4/VP7 genes.

**Conclusions:**

Dense, closed campus settings facilitate RVC clustered transmission. Limitations included absent screening of asymptomatic canteen staff. Rapid nucleic acid testing enabled timely pathogen identification for outbreak control. Greater attention should be paid to the public health risk of RVC. These whole-genome sequencing data enrich resources for studying RVC evolution and vaccine development.

## Highlights

• Reports a gastroenteritis outbreak by rotavirus C at a boarding high school in China.
• Obtains 13 complete RVC whole-genome sequences from a single outbreak.
• Genomic tracing and evolutionary analyses of RVC strains using global sequences.
• Multiplex PCR enables timely management of viral gastroenteritis outbreaks.

## Graphical abstract

**Figure:**
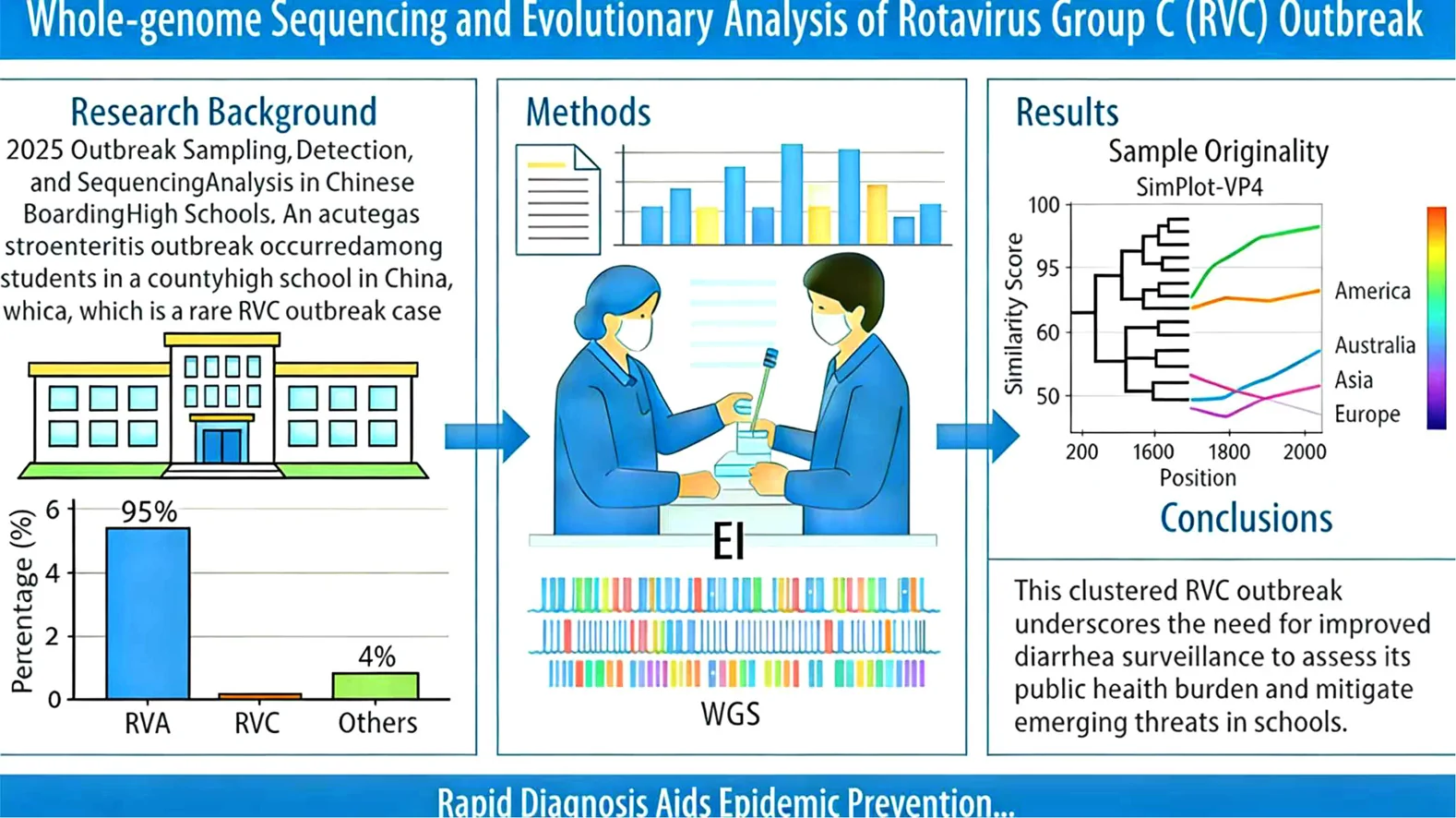

## 1 Introduction

Rotavirus is the leading etiological agent of diarrhea-associated mortality globally and a major cause of acute gastroenteritis among infants, young children, and neonates,^1^^,^^2^ with peak incidence observed during autumn and winter. Transmission occurs predominantly via the fecal-oral route and close person-to-person contact, making the virus highly capable of causing outbreaks in crowded, enclosed settings such as schools and childcare facilities.^3^ Upon infection, the virus primarily targets intestinal epithelial cells, in which its non-structural protein NSP4 acts as an enterotoxin that stimulates the enteric nervous system, thereby giving rise to typical clinical manifestations including diarrhea and vomiting.^4^ Vaccination continues to represent the cornerstone of rotavirus disease prevention and control.^5^

Rotavirus infection imposes a substantial disease burden in China. Surveillance data from 2009 to 2020 demonstrated that rotavirus infections accounted for 19.1% of all diarrhea cases,^6^ with associated socioeconomic costs reaching US$ 440 million in 2020 alone.^7^ Among human rotavirus infections, rotavirus A (RVA) is the predominant globally circulating genotype, accounting for over 95% of all strains, whereas rotavirus C (RVC) accounts for only approximately 1% of cases.^8^^,^^9^ Although human RVC infections can occur across all age groups, they remain predominantly sporadic.^10^ Global reports of human RVC infections are limited, with documented cases concentrated primarily in regions such as India, Republic of Korea, and China.^11^, ^12^, ^13^, ^14^

This report describes an outbreak of acute gastroenteritis among senior (final-year) students at a boarding high school in a county of Lu’an City, Anhui Province, China, in 2025. Comprehensive epidemiological investigation, rapid nucleic acid testing, and genomic tracing analysis confirmed that this incident was not only a rare RVC outbreak but also yielded an exceptionally large complete set of RVC whole-genome sequences (WGS) obtained from a single outbreak globally over the past 3 decades. These data provide a valuable resource for in-depth analysis of the genetic characteristics and evolutionary patterns of RVC.

## 2 Materials and methods

### 2.1 Epidemiological investigation

On May 16, 2025, multiple senior students at a boarding high school in Lu’an City, China, developed acute gastroenteritis characterized by diarrhea and vomiting. The County CDC immediately dispatched personnel to conduct a retrospective epidemiological investigation. Cases were defined as any student or staff member who experienced ≥ 1 episode of vomiting and/or ≥ 3 episodes of diarrhea with altered stool consistency within 24 h, with symptom onset on or after May 11, 2025. Case identification was performed through a systematic review of the school’s daily health monitoring records (morning and midday health checks), illness-related absenteeism logs, and visits to local medical institutions. As of May 19, a total of 33 suspected cases meeting the case definition were identified. Statistical analyses examined clinical symptoms, temporal distribution, population characteristics, dietary exposures, and potential risk factors.

### 2.2 Sample source

On May 16, 2025, the County CDC collected samples from final-year Grade 12 (Senior 3) students presenting with diarrhea and vomiting at the boarding school. A total of 19 samples were acquired, comprising 14 rectal swabs and 5 vomitus specimens. The present study was a retrospective investigation approved by the Ethics Review Committee of the Lu’an CDC (approval No. P2025-2531).

### 2.3 Polymerase chain reaction (PCR) detection of samples

Viral nucleic acids were extracted from all 19 samples using an automated nucleic acid extraction system (model Au1001-96; Wuxi Bio-Teke Biotechnology, Wuxi, China). Subsequently, quantitative real-time PCR was performed on a QuantStudio 5 instrument (Thermo Fisher Scientific, Walthum, USA) with an 8-plex nucleic acid detection kit for diarrheal pathogens (Beijing Applied Biological Technologies Co., Ltd., Beijing, China), which targets norovirus (GI and GII genotypes), RVA, rotavirus B (RVB), RVC, sapovirus, adenovirus, and astrovirus. All procedures were conducted in strict accordance with the manufacturer’s protocols, using reagents within their validated shelf life.

### 2.4 WGS and analysis

The 13 RVC-positive samples, designated with the “FX” prefix, were submitted to Beijing Macro-Micro Test Biotechnology for WGS. The sequencing protocol was executed as follows: libraries were prepared using a Rotavirus Whole-Genome Enrichment Kit (Probe Capture Method, Illumina version; catalog No. HWKF-TW0262; Jiangsu Macro-Micro Test Pharmaceutical Technology, Nantong, China). Libraries that passed quality control were sequenced using a PE150 platform on the Salus Por platform (Macro-Micro Test, Beijing, China). The raw sequencing data were processed with Fastp (version 0.23.4, OpenGene, Shenzhen, China.) to yield high-quality clean data. These clean reads were then aligned to 11 RVC reference sequences from the U.S. NCBI (National Center for Biotechnology Information) database (accession numbers: NC_007569; NC_007574; NC_007547; NC_007572; NC_007570; NC_007543; NC_007544; NC_007545; NC_007546; NC_007573; and NC_007571) using Minimap2 software (version 2.28-r1209, Broad Institute, Boston, USA) to generate binary alignment map files. Consensus sequences were derived using SAMtools (version 1.18, Wellcome Sanger Institute,Hinxton, UK), and variants were annotated with BCFtools (version 1.10.2, Wellcome Sanger Institute, Hinxton, UK). Final target sequences were produced following correction with custom Python scripts (maskregion_check.py and addNbase.py, developed in-house).

Multiple sequence alignment and phylogenetic tree construction: the VP7 and VP4 gene sequences from the 13 FX strains were aligned with those of known circulating RVC strains retrieved from the GenBank database using MEGA11 software (Molecular Evolutionary Genetics Analysis 11, Temple University, Philadelphia, USA). Phylogenetic trees were reconstructed based on the Minimum Evolution method^15^ and the robustness of the tree topology was assessed with 1,000 bootstrap replicates. Point mutation analysis and annotation: point mutations in the VP4 and VP7 sequences of the 13 FX strains were identified by comparison against the NCBI-recommended RVC reference genome (NC_007572.1; NC_007543.1) using Snippy (version 4.6.0, University of Vienna, Vienna, Austria). The identified variants were subsequently annotated for their potential effects using (version 4.6.0, University of Vienna, Vienna, Austria). Segment reassortment analysis: to investigate segment reassortment, all 11 gene segments from the 13 FX strains were analyzed using BLAST alignment against the comprehensive set of RVC sequences available in the NCBI database. Recombination analysis and verification: recombination events within the VP4 and VP7 sequence sets were investigated using RDP5 (version 5.57, University of Cape Town, Cape Town, South Africa). The analysis involved comparisons with CDS data sets of human-origin RVC VP4 and VP7 from various global regions (including Asia, Europe, North America, and Australia) obtained from the NCBI database. Detection was performed using multiple algorithms (RDP, GENECONV, BootScan, 3Seq, LARD), and the identified recombination signals were validated using SimPlot software (version 5.57, Johns Hopkins University, Baltimore, USA).

## 3 Results

### 3.1 Epidemiological investigation results

#### 3.1.1 Temporal, population, and dietary exposure distribution of cases

**Fig. 1:**
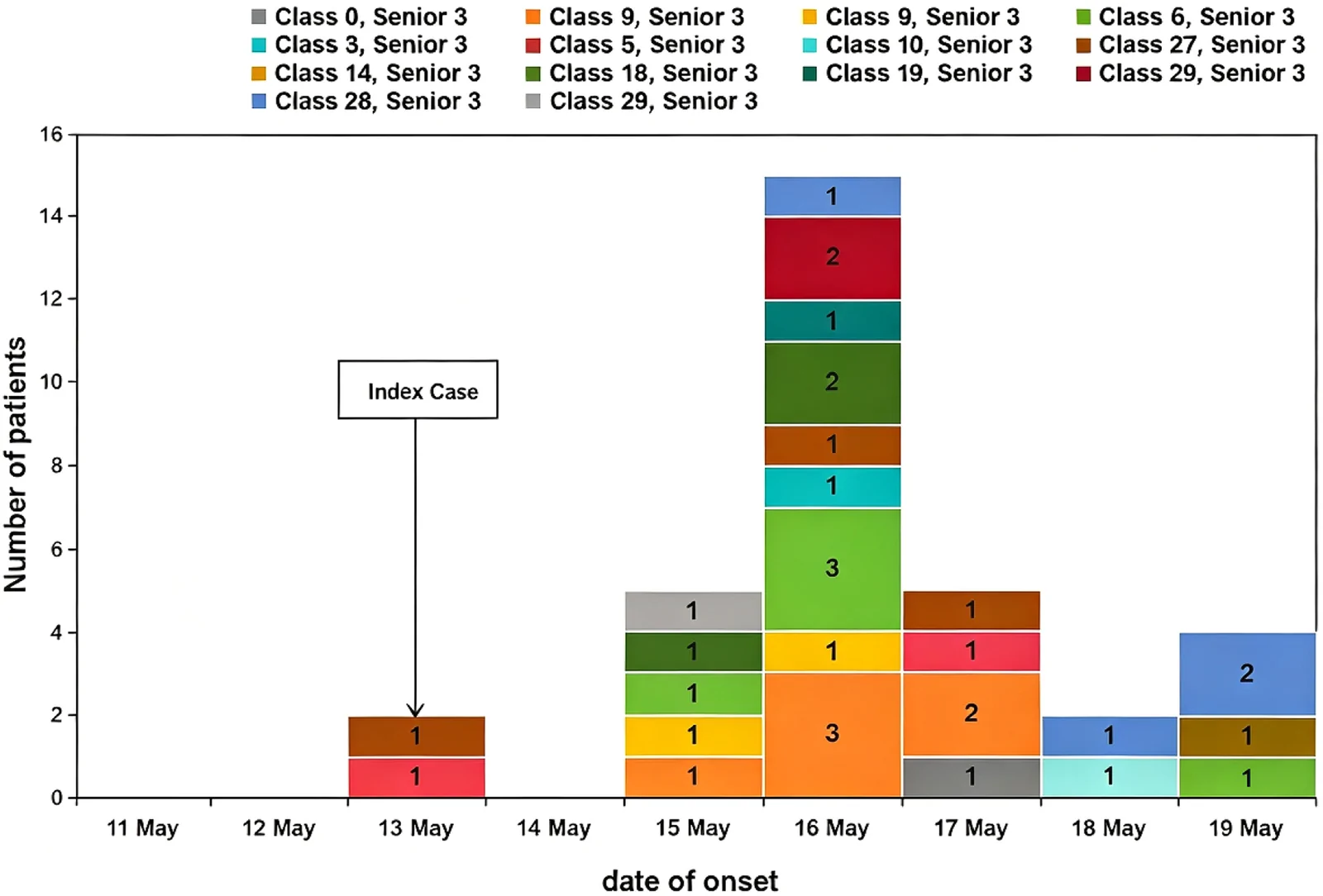
Temporal distribution of the number of acute gastroenteritis cases in a high school. *Note*: Numbers in the colored boxes indicate the number of cases.

Epidemiological investigation demonstrated that all cases in this outbreak were among Senior 3 students. The school had 29 Senior 3 classes with a total enrollment of 1453 students. During the outbreak, 33 suspected cases were identified, accounting for 2.3% of the total Senior 3 student population (33/1453). The index case had symptom onset on May 13, while most cases occurred on May 16 and were scattered across 14 Senior 3 classes, including in 22 male and 11 female students. Details are presented in Fig. 1. All students and staff of the school dined in the same cafeteria daily; drinking water was provided as bottled purified water, which was heated using water dispensers before consumption.

#### 3.1.2 Clinical characteristics of cases

Among the 33 cases meeting the case definition, diarrhea and vomiting represented the predominant clinical manifestations, occurring in 69.7% and 42.4% of cases, respectively. Secondary symptoms included fever (≥ 37.3°C), abdominal pain, and nausea, with incidences of 15.2%, 15.2%, and 6.1%, respectively.

### 3.2 Results of 8-plex diarrheal virus PCR assay

The 8-plex diarrheal virus PCR assay detected RVC in 11 of 14 rectal swabs and 2 of 5 vomitus samples. All samples tested negative for the other 7 target viruses, including norovirus GI and GII genotypes, RVA, rotavirus B, sapovirus, adenovirus, and astrovirus. The overall RVC detection rate was 68.4% (13/19).

### 3.3 Genetic evolution and variation analysis

#### 3.3.1 Phylogenetic analysis of VP7 and VP4 genes

**Fig. 2:**
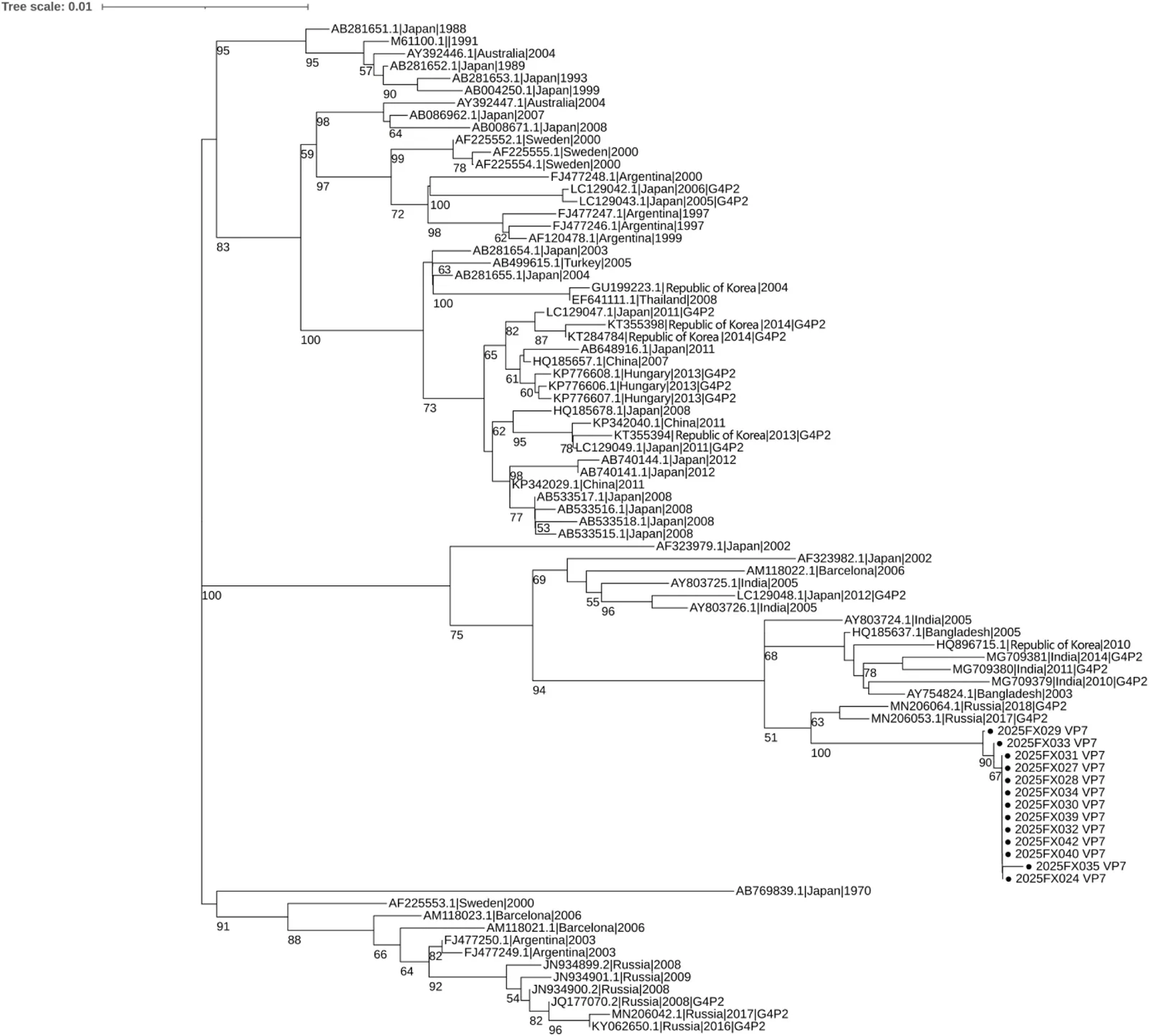
Phylogenetic tree of VP7 genes. phylogenetic trees of VP7 sequences from the FX strains and homologous RVC epidemic and reference strains retrieved from the GenBank database, constructed using the minimum evolution method in MEGA X. Notes: Black circles represent the 13 FX strains in this study. Numbers at nodes (above 50%) indicate bootstrap support (%). Scale bar, genetic distance.

**Fig. 3:**
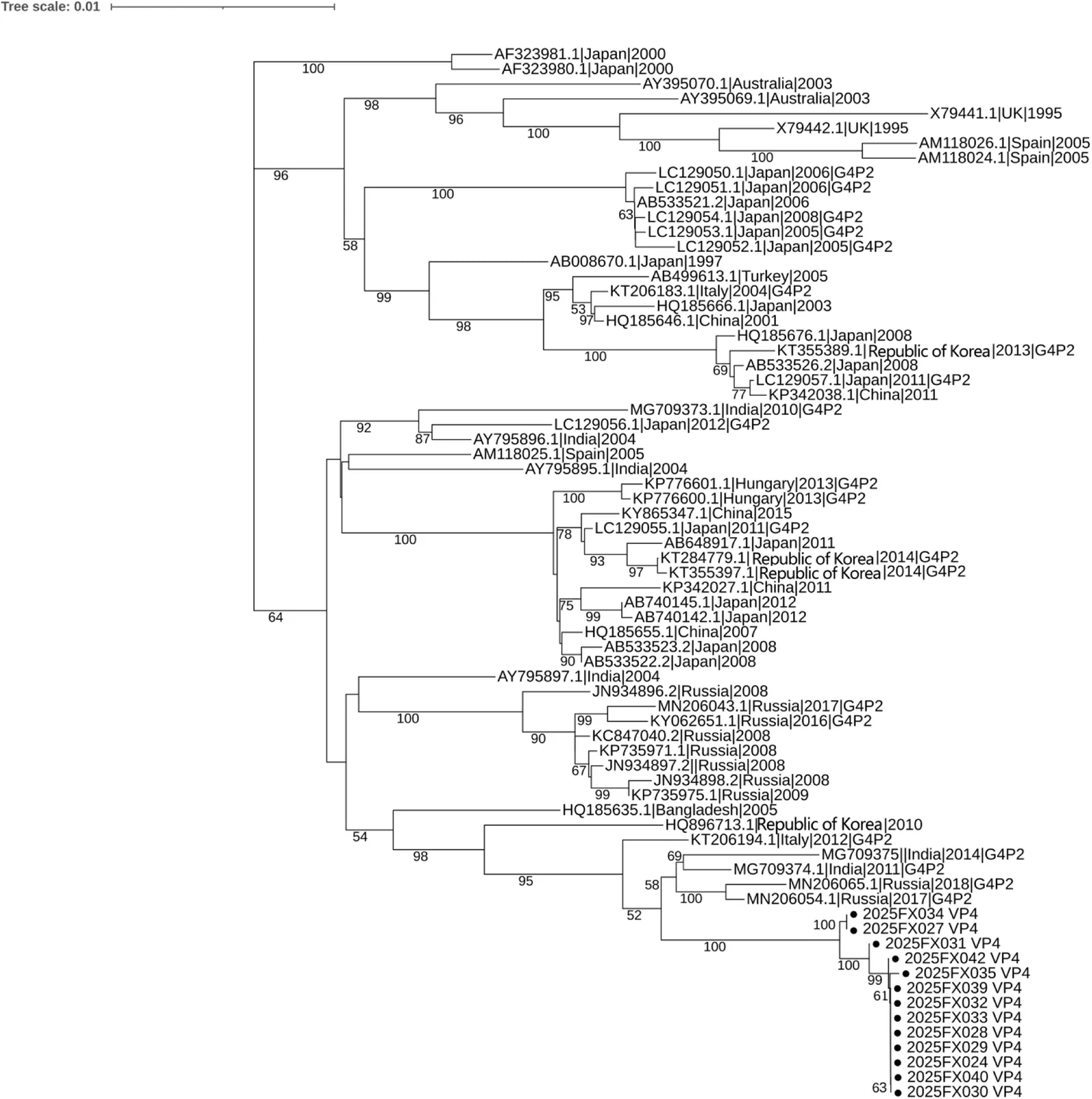
Phylogenetic tree of VP4 genes. Phylogenetic trees of VP4 sequences from the FX strains and homologous genes of RVC epidemic and reference strains retrieved from the GenBank database, constructed using the minimum evolution method in MEGA X. Notes: Black circles represent the 13 FX strains in this study. Numbers at nodes (above 50%) indicate bootstrap support (%). Scale bar, genetic distance.

Phylogenetic analysis of the VP7 and VP4 genes was conducted by comparing the 13 FX strains with known circulating RVC strains deposited in the GenBank database using MEGA11 software. Multiple sequence alignment demonstrated that the VP7 and VP4 sequences of the 13 RVC FX strains from this outbreak clustered into a highly conserved monophyletic clade (Figs. 2 and 3). These strains exhibited remarkably high nucleotide sequence similarity, with within-group homology values ranging from 99.3% to 100.0% for both genes.

##### 3.3.1.1 VP7 gene phylogeny

The 13 FX strains demonstrated the closest genetic relationship with Russian strains isolated in 2017 (MN206053.1) and 2018 (MN206064.1), with a sequence similarity of 98.1–98.6% for both gene segments. High similarity was also observed with Asian strains, including those from Japan in 2005 (HQ185637.1, 97.7–98.2%), India in 2005 (AY803724.1, 97.7–97.9%), and Republic of Korea in 2010 (HQ896715.1, 97.5–97.8%). By contrast, comparisons with previously reported Chinese strains revealed lower similarity: 94.5–94.7% with the 2011 strain (KP342029.1), 94.4–94.6% with the 2007 strain (HQ185657.1), and 94.1–94.4% with another 2011 strain (KP342040.1).

##### 3.3.1.2 VP4 gene phylogeny

The 13 FX strains exhibited the highest sequence similarity to the 2017 Russian strain (MN206054.1, 98.4–98.9%) and the 2018 Russian strain (MN206065.1, 98.2–98.7%). Additional closely related strains included those from Italy in 2012 (KT206194.1, 97.4–98.0%), Republic of Korea in 2010 (HQ896713.1, 96.9–97.5%), and Japan in 2005 (HQ185635.1, 96.7–97.2%). Comparisons with Chinese strains yielded similarity values of 96.1–96.6% for the 2007 strain (HQ185657.1), 96.0–96.5% for the 2015 strain (KY865347.1), and 95.8–96.3% and 95.3–95.8% for two 2011 strains (KP342027.1 and KP342038.1, respectively).

#### 3.3.2 Point mutation analysis

Point mutation analysis of the VP4 and VP7 sequences from the 13 FX strains revealed distinct mutational patterns. For the VP4 gene, each strain harbored 26 amino acid variant sites, including a single threonine deletion (c.744_747delAACTinsA) classified as a disruptive_inframe_deletion, a variant type characterized by the removal of one or more bases from the DNA sequence without disrupting the reading frame. By contrast, the VP7 gene exhibited 7 amino acid variant sites per strain, with no disruptive_inframe_deletion variants detected. Comprehensive variant annotations and detailed results are available in the Supplementary Table S1.

#### 3.3.3 Segment reassortment analysis

BLAST alignment of all 11 gene segments from the 13 FX strains against the complete RVC sequence database in NCBI demonstrated consistently high homology with the Italian PR713 strain (KT206197.1), with sequence similarities ranging from 97.7% to 99.1%. Despite these minor variations in individual segment similarities, the overall homology patterns provided no evidence of segment reassortment events among the FX strains.

#### 3.3.4 Genetic recombination analysis

**Fig. 4:**
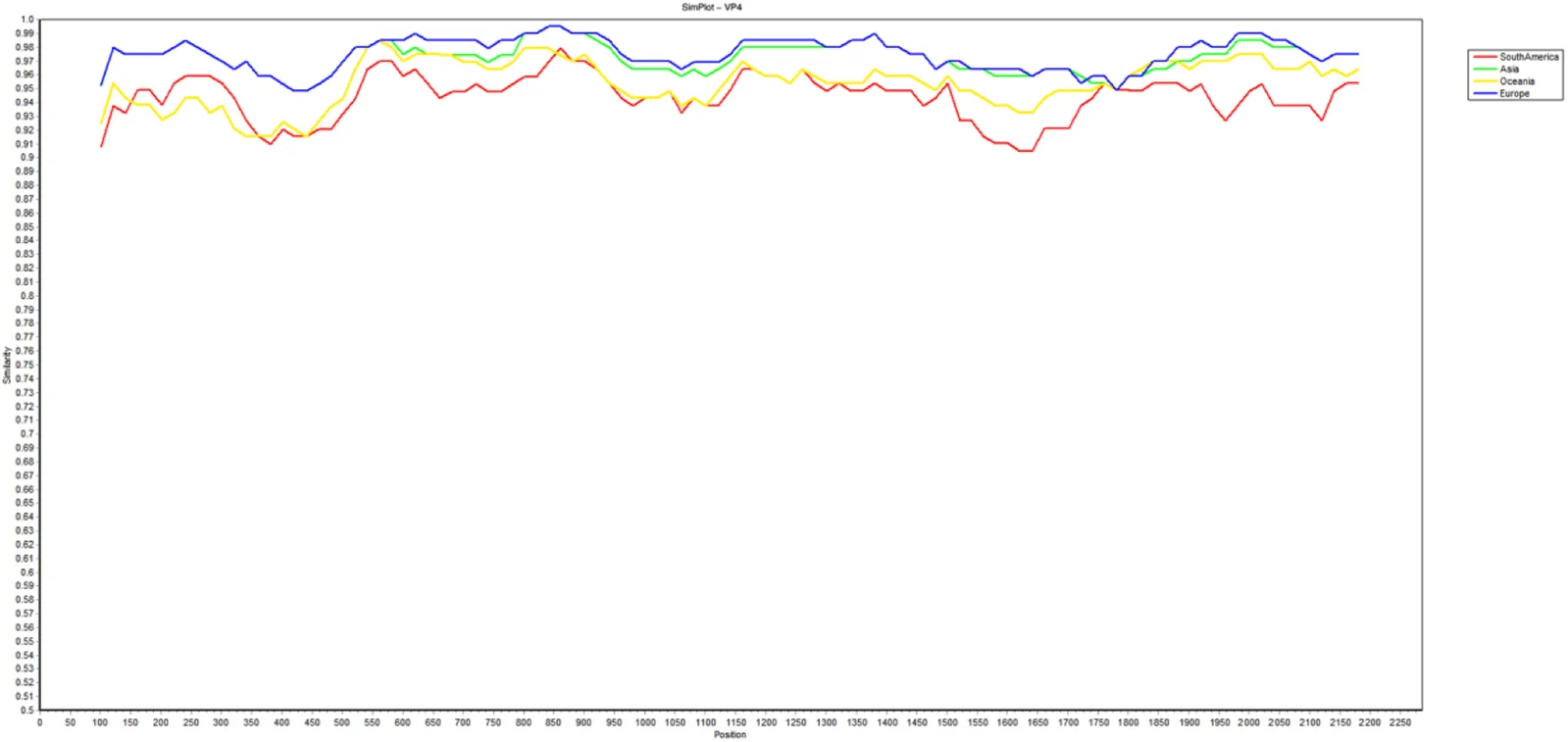
SimPlot recombination analysis of the VP4 gene in the 13 FX strains. Detection of possible recombination events in the 13 FX strains. Notes: Recombination events of the 13 FX strains were analyzed using the recombination detection software SimPlot and RDP (version 5.57). Complete CDS sequences of the VP4 genes of RVC from regions including South America (red), Asia (green), Oceania (yellow), and Europe (blue) were used as candidate parental sequences. The vertical axis represents pairwise sequence identity between the recombinant strains and candidate parental strains, whereas the horizontal axis indicates the nucleotide positions of the aligned sequences. Abbreviation: VP, viral protein.

**Fig. 5:**
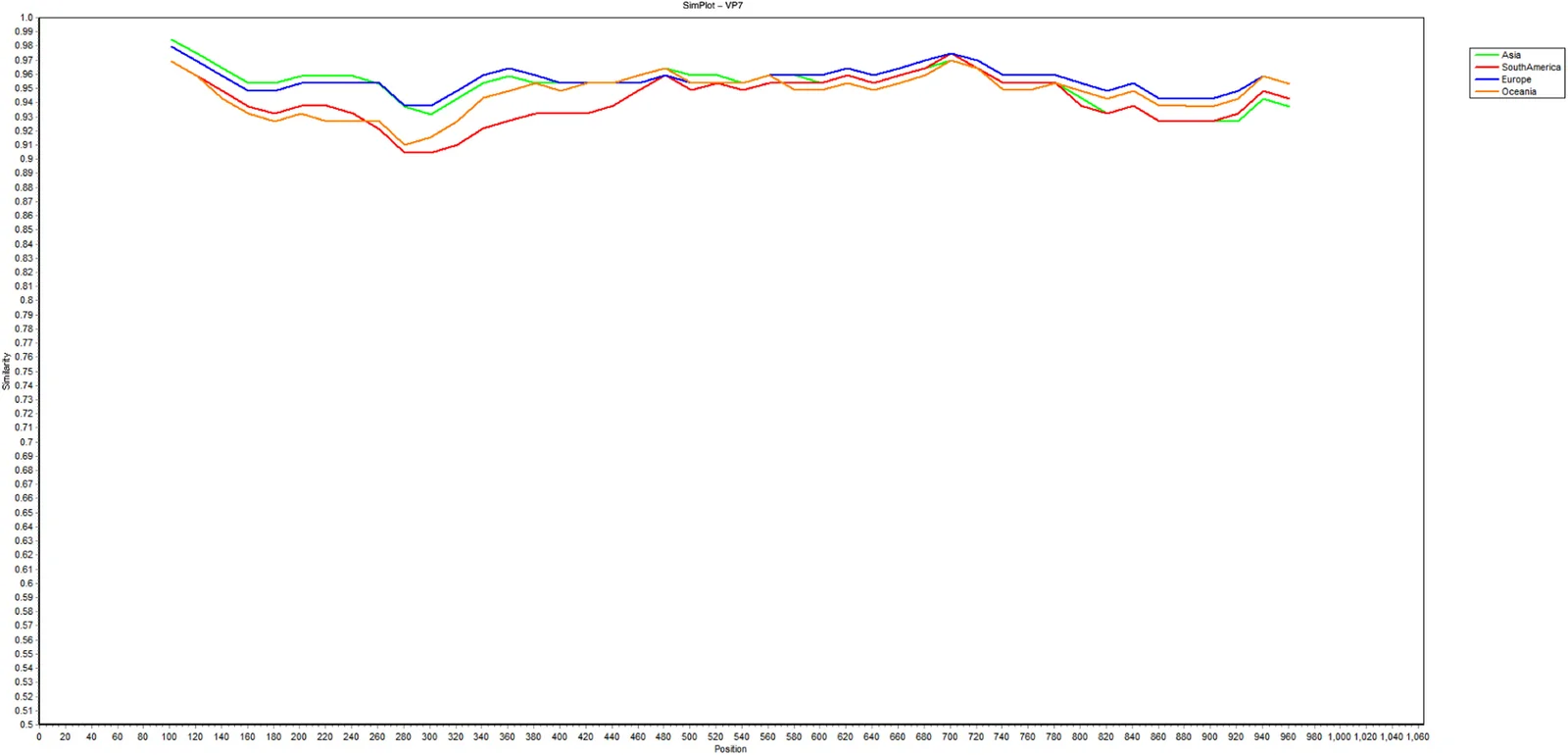
SimPlot recombination analysis of the VP7 gene in the 13 FX strains. Detection of possible recombination events in the 13 FX strains. Notes: Recombination events of the 13 FX strains were analyzed using the recombination detection software SimPlot and RDP (version 5.57). Complete CDS sequences of theVP7 genes of RVC from regions including South America (red), Asia (green), Oceania (yellow), and Europe (blue) were used as candidate parental sequences. The vertical axis represents pairwise sequence identity between the recombinant strains and candidate parental strains, whereas the horizontal axis indicates the nucleotide positions of the aligned sequences. Abbreviation: VP, viral protein.

To assess potential recombination events, we compared the 13 FX strain sequences with CDSs of the VP4 and VP7 genes from RVC strains isolated across Asia, Europe, South America, Oceania, and other regions available in the NCBI database. This recombination analysis revealed no significant recombination events. Independent validation confirmed the absence of recombination in both the VP4 and VP7 genes (see Figs. 4 and 5).

## 4 Discussion

This study identified an uncommon acute gastroenteritis outbreak caused by RVC in a boarding high school located in Lu’an City, China. By integrating epidemiological characteristics, etiological detection, and whole-genome phylogenetic analysis, we systematically investigated the transmission patterns, viral genetic origin, and molecular evolutionary features of this outbreak, thereby providing critical insights for the prevention, control, and surveillance of RVC-associated outbreaks.

The outbreak exhibited typical characteristics of campus clustering, with cases exclusively confined to Senior 3 students and no reported cases among faculty members. Given that students and staff shared the same canteen and bottled drinking water supply, the possibility of foodborne or waterborne transmission was considered low. Temporally, the onset of cases peaked around May 16, with the index case occurring on May 13, which was highly consistent with the typical incubation period of RVC (2–3 days). Epidemiological evidence suggested that a large-scale indoor gathering of Senior 3 students on May 11 might have served as the key exposure event. The densely populated and poorly ventilated environment significantly facilitated viral transmission via vomit aerosols and close contact. At the same time, fecal-oral transmission mediated by contaminated environmental surfaces also posed a substantial risk. This observation is consistent with the well-documented tendency of RVC to cause point-source outbreaks in confined populations, justifying the definition of cases as those with symptom onset on or after May 11, 2025. Congregate settings such as schools and medical institutions are prone to RVC outbreaks. Previous studies have reported RVC outbreaks among neonates in hospital wards in Republic of Korea^16^ and an earlier outbreak involving primary school students and staff was documented in Fukui City, Japan, in 1988.^17^

Etiological testing revealed an overall RVC positivity rate of 68.4% among 19 clinical specimens, with the exclusion of the 7 other common diarrheal viruses (including norovirus and RVA), providing direct evidence to confirm the etiological agent. The negative results in a small number of samples were likely attributed to delayed sampling, reduced viral load, or inefficient nucleic acid extraction, and mixed infections with multiple pathogens were largely ruled out. Consistency between epidemiological evidence and laboratory findings confirmed that this outbreak was caused by a single RVC strain.

Phylogenetic analysis of the whole-genome sequences showed that the 13 FX strains obtained in this study exhibited high intra-strain homology (99.3–100.0%) in the VP7 and VP4 genes and formed a distinct monophyletic clade, indicating a single source of infection rather than multiple introductions or repeated transmission events. Phylogenetic relationships demonstrated that the FX strains shared the closest genetic relationship with RVC strains circulating in Russia during 2017–2018 (nucleotide homology > 98.0%), followed by strains from Japan, India, Republic of Korea, and other Asian countries. By contrast, they showed a relatively distant genetic relationship with previously reported RVC strains in China (homology ≤ 94.7%). These results suggest that the circulating strain in this outbreak did not evolve from indigenous Chinese strains but was more likely introduced from overseas lineages, which further highlights the insufficient attention paid to the cross-regional importation risk of RVC in China.

At the molecular variation level, all FX strains in this study exhibited 26 amino acid variations in VP4, with a complex insertion-deletion variant associated with threonine deletion (c.744_747delAACTinsA) identified in the key antigenic region downstream of the trypsin cleavage site. This variant is an in-frame variant, characterized by a 4-bp deletion (AACT) accompanied by a 1-bp insertion (A), leading to a net deletion of 3 nucleotides. Functionally, this variant is equivalent to the deletion of a single threonine residue at amino acid position 248, which corresponds to the N-terminal region of VP5. Based on the localization of this site within a conserved region and published literature regarding the structure of rotavirus VP4, we speculate that this variant may affect viral membrane fusion capacity and antigenic epitope conformation, thereby posing potential risks of immune escape and antigenic alterations.^9^ Additionally, this variant site is located immediately downstream of the VP8 trypsin cleavage site (aa241), suggesting that it may also impact the spatial structure of the VP4 spike protein and consequently reduce viral sensitivity to trypsin.^18^ It should be noted that the function-related speculations require further validation through conservation sequence analysis and site-directed mutagenesis experiments.

Notably, this study obtained 13 complete RVC genome sequences from a single outbreak, representing one of the largest datasets of RVC genomes from a single outbreak globally over the past 3 decades. This dataset significantly enriches the molecular epidemiological data available for RVC in China. Currently, surveillance data and genome sequence accumulation for RVC in China are scarce, making it difficult to track epidemic lineages and variation trends accurately. This outbreak also exposed obvious deficiencies in the routine surveillance of RVC in high-density populations such as students. Traditionally regarded as a sporadic pathogen, the potential of RVC to cause large-scale outbreaks in confined settings (e.g., schools) has not been fully recognized. This potential highlights the need to include RVC in routine screening for diarrheal syndromes, rather than focusing solely on RVA and norovirus.

To date, there is no licensed RVC vaccine worldwide, and research on key scientific issues such as antigenic variation and immune escape of RVC remains limited. The key deletion mutation in VP4 identified in this study provides important information for future studies on RVC antigenicity and the screening of candidate vaccine targets. Meanwhile, the application of rapid sampling and multiplex PCR detection in the response to this outbreak provided strong support for the timely identification of the pathogen and the implementation of targeted prevention and control measures, confirming the crucial role of rapid molecular detection techniques in the emergency response to viral gastroenteritis in school settings.

This study has several limitations. First, the scope and depth of the epidemiological investigation were limited. Tracing of case exposure histories and investigation of high-risk sites were not sufficiently detailed, and cross-validation of exposure information through multiple channels was not performed. For instance, although no clinical symptoms were reported among faculty members, asymptomatic infection screening was not conducted for canteen staff responsible for the senior high school section, making it impossible to completely rule out the possibility of contamination by asymptomatic food handlers. Second, the source-tracing investigation was not systematic, failing to accurately identify the source of infection, contamination route, and key driving factors of the outbreak. Additionally, due to the absence of a licensed RVC vaccine, it was not feasible to compare the VP4 and VP7 sequences of wild-type strains with reference strains(as conducted in studies on RVA.^19^^,^^20^) to analyze antigenic differences and potential immune escape risks. Furthermore, among the 33 students who met the case definition, some had already recovered from their symptoms or refused to provide samples, resulting in the collection of only 19 specimens, which limited the comprehensiveness of the analysis to a certain extent.

## 5 Conclusion

RVC can cause large-scale acute gastroenteritis outbreaks in high-density populations such as schools, and its disease burden and public health risk require re-evaluation. In the future, systematic surveillance and WGS of RVC should be strengthened to monitor its epidemic lineages and genetic variation characteristics dynamically, improve early identification and emergency response capabilities, and prevent the recurrence of similar clustered outbreaks.

## CRediT authorship contribution statement

**Dawei Gao:** Writing – original draft, Methodology, Data curation, Conceptualization. **Zhichao Chen:** Resources, Methodology, Investigation, Data curation. **Shunyi Zhang:** Methodology, Investigation, Data curation. **Lulu Zhang:** Supervision, Methodology, Data curation. **Hongwei Chang:** Writing – review & editing, Supervision. **Junli Chen:** Resources, Methodology, Investigation. **Yuanyuan Shao:** Resources, Investigation. **Shukun Yu:** Methodology, Data curation. **Chong Liu:** Supervision, Methodology, Data curation.

## Informed consent

Written informed consent was obtained from patients for the publication of data and figures related to this study.

## Organ donation

This study did not involve organ donation or any relevant procedures.

## Ethics statement

This study was ethically reviewed and approved by the Lu’an CDC and Huoshan County CDC Joint Ethics Committee under approval number P2025-2531. The approval covered sample testing, genetic tracing and epidemiological investigation of the gastroenteritis outbreak. All experimental and investigative procedures were carried out in strict compliance with the approved ethical protocols.

## Data availability statement

All data generated or analyzed during this study are included in this published article. Other original data supporting these findings can be obtained from Dawei Gao upon reasonable request. The detected gene sequences have been submitted to the GenBank database with accession numbers PV870536–PV870561.

## Animal treatment

No animal experiments were performed in this study.

## Generative AI

Generative AI was used for language polishing only. All content was carefully reviewed, revised and verified by the authors, who take full responsibility for the integrity and accuracy of the manuscript.

## Funding

This research received no specific grant from any funding agency.

## Declaration of competing interest

The authors declare that they have no known competing financial interests or personal relationships that could have appeared to influence the work reported in this paper.

The author is an Editorial Board Member/Editor-in-Chief/Associate Editor/Guest Editor for this journal and was not involved in the editorial review or the decision to publish this article.

## Appendix Supplementary materials

Image, application 1
